# The Absorption, Distribution, Excretion, and In Vitro Hepatic Microsomal Metabolism of the Novel CDK Compound XMD12 in Sprague-Dawley Rats

**DOI:** 10.3390/pharmaceutics17121545

**Published:** 2025-11-30

**Authors:** Xue-Hai Zheng, Yan-Chun Chang, Yong-Hui Li, Yu-Xia He, Pei-Xiong Zhao, Fei-Fei Wang, Jun-Yu Xu, Yin-Feng Tan

**Affiliations:** 1 School of Pharmacy, Hainan Medical University, 3 Xueyuan Road, Haikou 571159, China; xuehaihaihai@gmail.com (X.-H.Z.); 18419870189@163.com (Y.-C.C.); lyhssl@126.com (Y.-H.L.); 15738088269@163.com (Y.-X.H.); zhaopeixiong0824@163.com (P.-X.Z.); 15799016982@163.com (F.-F.W.); 2 Engineering Research Center of Tropical Medicine Innovation and Transformation of Ministry of Education, Haikou 571199, China; 3 Hainan Key Laboratory for Research and Development of Tropical Herbs, School of Pharmacy, Hainan Medical University, Haikou 571199, China; 4 Haikou Key Laboratory of Li Nationality Medicine, Haikou 571199, China

**Keywords:** CDK4/6 inhibitors, LC–MS/MS, method validation, antitumor, cell cycle, selectivity

## Abstract

**Background**: Cyclin-dependent kinase 4/6 (CDK4/6) represents one of the clinically validated and therapeutically effective anticancer targets. **Methods**: This study established a rapid and sensitive HPLC-MS/MS method for quantitative determination of XMD12, a novel CDK4/6 inhibitor developed in our laboratory, in various rat biological matrices including plasma, tissue homogenates, urine and feces. The experimental design comprised three distinct phases: pharmacokinetic evaluation in 10 Sprague-Dawley SD rats (5 receiving 2.0 mg/kg intravenous injection via tail vein and 5 administered 10.0 mg/kg by gastric gavage); tissue distribution assessment in 25 SD rats following a single 20 mg/kg gastric gavage; and an excretion study in 5 SD rats following a single 20 mg/kg gastric gavage. Biological samples were systematically collected post-dosing and analyzed using a validated LC-MS/MS method. **Results**: Key pharmacokinetic characteristics revealed (1) delayed absorption with significantly limited systemic exposure following gastric gavage; (2) preferential hepatic accumulation post-intestinal absorption followed by rapid clearance through metabolic/biliary pathways with multi-organ collaborative elimination; and (3) time-dependent cumulative excretion predominantly via feces, suggesting final clearance through biliary-mediated intestinal elimination of metabolites. **Conclusions**: These findings demonstrate XMD12’s dynamic disposition pattern characterized by “restricted absorption–hepatic metabolic priority–multi-tissue collaborative clearance” in vivo. This comprehensive investigation provides crucial pharmacokinetic parameters and excretion profiles for the anticancer lead compound XMD12, offering valuable insights for pharmacodynamic and toxicological evaluations while establishing a foundation for structural optimization and derivative screening of lead compounds.

## 1. Introduction

CDK4/6 inhibitors play a vital role in precision medicine by enabling personalized treatment strategies based on individual patient characteristics such as genomic and mutation profiles [1]. These agents are particularly significant in treating malignancies like breast cancer and small-cell lung cancer [2,3,4], markedly improving both median progression-free survival and overall survival [5], and demonstrating breakthrough potential. In recent years, several CDK4/6 inhibitors, including Palbociclib, ribociclib, and abemaciclib, have been approved by the FDA for use in combination with aromatase inhibitors or fulvestrant as initial treatment for hormone receptor-positive, HER2-negative advanced or metastatic breast cancer [6,7,8]. Underlying these clinical benefits are significant differences in pharmacokinetic behavior, reflecting the structural diversity of their chemical scaffolds. While sharing the same molecular target, the approved CDK4/6 inhibitors exhibit distinct pharmacokinetic profiles and clinical characteristics arising from their diverse chemical scaffolds. These well-established pharmacokinetic properties provide a valuable framework for evaluating new candidates, while simultaneously revealing significant opportunities to expand the chemical diversity of this drug class through novel scaffolds with optimized ADME characteristics [9]. Against this background, systematic pharmacokinetic evaluation serves as the critical first step in assessing new molecular entities.

Pharmacokinetic (PK) studies are essential in early drug development, providing critical information on the absorption, distribution, metabolism, and excretion of candidate compounds, which helps identify molecules with favorable PK properties and improves the likelihood of successful drug development [10]. The well-documented PK profiles of established CDK4/6 inhibitors—such as bioavailability, excretion routes, and tissue distribution—serve as an important reference for screening and evaluating new drug candidates [11,12,13]. Existing inhibitors exhibit notable PK similarities: Palbociclib, Ribociclib, and Abemaciclib have moderate oral bioavailability (46%, 65.8%, and 45%, respectively), are rapidly absorbed (with Tmax values within 6–12, 1–5, and under 8 h, respectively), and have prolonged elimination half-lives (24–34, 30–55, and 17–38 h, respectively). They are primarily metabolized by CYP3A4 in the liver and excreted via feces. Palbociclib shows a large volume of distribution (∼2800 L) and 85.3% plasma protein binding, while Abemaciclib has 96.3% plasma protein binding [14,15,16]. Predicting drug-likeness relies heavily on PK evaluation. Using Abemaciclib as a lead structure, we designed and synthesized a novel series of compounds through rational approaches such as scaffold hopping. Among these, the candidate XMD12 shows promising structure-activity relationships and development potential, possibly expanding our understanding of CDK4/6 inhibitors and offering new therapeutic options. However, its PK properties remain entirely unknown, making systematic evaluation a priority for subsequent research.

This study establishes a rapid and sensitive HPLC-MS/MS method for quantifying XMD12—a novel CDK4/6 inhibitor developed in our lab—in various rat biological matrices (plasma, tissue homogenates, urine, and feces), providing a foundation for subsequent quantitative analysis of such compounds. Determining key PK parameters, tissue distribution, and excretion profiles will help reveal the in vivo behavior of the anticancer lead compound XMD12 in rats, offer important clues for pharmacodynamic and toxicological evaluation, and support further structural optimization and derivative screening. This work will also aid in identifying target organs related to efficacy or toxicity, clarify the material basis of its effects, and provide critical insights for developing safer and more effective new drugs as well as potential detoxification strategies.

## 2. Materials and Methods

### 2.1. Chemical Materials and Reagents

Compounds XMD12 and the internal standard (IS) XMD14 were obtained in our laboratory [17]. Their structures were elucidated using nuclear magnetic resonance and mass spectrometry, and purity (≥98%) was determined using high-performance liquid chromatography (HPLC) (Figures S1 and S2), as detailed in the Supplementary Material. Isoflurane (No. 201808) was purchased from Gene & I Scientific Co., Ltd. (Beijing, China) and HPLC-grade methanol and formic acid were purchased from Merck (Darmstadt, Germany); ultrapure water was used.

### 2.2. Animal Studies

All experimental procedures involving animals were conducted in compliance with protocols approved by the Institutional Animal Care and Use Committee of Hainan Medical University (Haikou, China). Female Sprague-Dawley (SD) rats (body weight range: 220–280 g) were sourced from Changsha Tianqin Bio-technology Co., Ltd. (Changsha, China; Certification No. SCXK2022–0011). The animals were housed under controlled environmental conditions, maintaining a temperature of 20–24 °C, relative humidity of 40–70%, and a standardized 12-h light/dark cycle. Standard laboratory diet was provided ad libitum, interrupted only by an overnight fast preceding drug administration. Throughout the study, all rats had unrestricted access to water.

### 2.3. Instrumentation and Analytical Conditions

Chromatographic separation and mass spectrometric detection were performed using an AB-SCIEX API 4000 plus triple quadrupole mass spectrometer (Toronto, ON, Canada) coupled with a Shimadzu Nexera XR ultra high-performance liquid chromatographic system (Kyoto, Japan). The HPLC system was equipped with two LC-20AD pumps, a DGU-20A3R degassing unit, a SIL-20A HT autosampler, and a CTO-20A column oven. System control, data acquisition, and processing were managed by AB-SCIEX Analyst 1.6.2 software.

Separation of analytes was achieved on a Phenomenex Kinetex XB-C18 column (2.10 mm × 50 mm, 2.6 µm) maintained at 40 °C. The mobile phase comprised solvent A (0.03% formic acid in water) and solvent B (acetonitrile), using the following gradient program: 20% B (0–0.5 min), increased to 95% B (0.5–2.0 min), held at 95% B (2.0–3.5 min), returned to 20% B (3.5–4.0 min), and re-equilibrated at 20% B (4.0–5.0 min). The flow rate and injection volume were set at 0.4 mL/min and 3 µL, respectively.

The mass spectrometer was operated in positive ESI ion mode with selected multiple reaction monitoring (MRM) mode for XMD-12 and IS (XMD-14). The spray voltage was 5.0 kV, and the desolvation temperature was 550 °C. The pressure of the inner coaxial nebulizer N2 gas (GS1), dry N2 gas (GS2), curtain N2 gas (CUR) and collision gas (CAD) was 50 psi, 50 psi, 35 psi and grade 2, respectively. The ion pairs of XMD-12 and IS were *m*/*z* 399.1 → 353.1 (declustering potential 124 eV; collision energy 45 eV) and *m*/*z* 416.2→382.0 (declustering potential 127 eV; collision energy 54 eV), respectively, and dwell time of 50 ms.

### 2.4. Preparation of Standard and Quality Control Samples

Accurately weigh XMD12 with precision and craft a stock solution at a concentration of 1 mg/mL in acetonitrile (10 mL), securely storing it in a refrigerator at 4 °C for future use. Gradually dilute an appropriate volume of the 1 mg/mL XMD12 stock solution with pure acetonitrile to produce intermediate working standard solutions at concentrations of 10, 100, 500, 1000, 10,000, and 20,000 ng/mL, respectively. Precisely measure the standard substance XMD14, and dilute it with acetonitrile to create a 1 mg/mL internal standard stock solution, which should also be kept in the refrigerator at 4 °C for later utilization. Before usage, dilute the internal standard stock solution with acetonitrile to a concentration of 150 ng/mL. For the preparation of calibration standards, combine 5 µL of the suitable standard solution, 150 µL of the internal standard stock solution, and 50 µL of blank rat plasma, urine, feces, or tissue homogenate samples, producing the final calibration standards with target concentrations of 10, 100, 500, 1000, 10,000, and 20,000 ng/mL, respectively. Quality control (QC) samples encompass the lower limit of quantification (LLOQ), low, medium, and high QC levels. These are prepared in the same manner, utilizing plasma, urine, feces, and tissue samples, yielding QC samples with final target concentrations of 50, 900, and 16,000 ng/mL. Both standard and QC samples are to be stored at 4 °C until the time of analysis.

### 2.5. Sample Preparation

Both urine, fecal, and tissue samples need to be measured for mass or volume. Plasma and urine samples can be directly collected and processed. When dealing with fecal samples, they should be finely ground in a mortar and pestle, then mixed with four times their mass in deionized water and thoroughly homogenized within an ice bath. As for tissue samples, once they’re cut into smaller sections, they should be blended with three times their mass in saline solution, using an ice bath to ensure proper homogenization. Subsequently, take 50 μL of plasma, urine, or the homogenized fecal and tissue samples, combine them with 150 μL of the internal standard stock solution, shake vigorously for 10 min, and then centrifuge at 12,000 rpm for 10 min at 4 °C. Finally, collect the supernatant for subsequent LC-MS/MS analysis. For accurate quantification, the concentration obtained from the calibration curve was accordingly multiplied by the respective dilution factor to back-calculate the original concentration in the neat sample.

### 2.6. Method Validation

Our analytical approach adheres to a comprehensive set of validation procedures in accordance with the recommendations outlined in the U.S. Food and Drug Administration’s Bioanalytical Method Validation Guidance (USFDA, 2018) [18]. These validation steps encompass a range of critical factors, including method specificity, linearity, sensitivity, carryover effect, accuracy, precision, matrix effect, recovery, dilution integrity, and stability. In assessing the method’s selectivity, we initiated the process by comparing chromatograms obtained from various sample sources. These sources included blank rat plasma, blank rat serum samples spiked with a mixture of standards, and rat plasma samples sourced from pharmacokinetic studies conducted on rats. This evaluation concurrently aided us in identifying and addressing any potential interferences that may occur within the acquisition window of XMD12 and the internal standard (IS). To evaluate linearity, a series of XMD12 concentrations was introduced into blank plasma, allowing us to establish a linear regression equation. The calibration curve (n = 5) was constructed by plotting the ratio of peak areas of XMD12 to IS (y-axis) against the nominal concentration ratio of XMD12 to IS (x-axis), followed by subsequent linear regression analysis. The lower limit of quantification (LLOQ) was defined as the lowest concentration point on the calibration curve that guarantees reliable quantification while maintaining accuracy within the range of 80–120% and precision below 20%. We conducted assessments of intra-day and inter-day precision and accuracy by performing six replicate tests on quality control (QC) samples at concentrations of 3, 120, and 1500 ng/mL over both a single day and a span of three days. Matrix effects and extraction recovery were evaluated through a post-extraction addition approach. Furthermore, we conducted a study on the stability of QC samples with high, medium, and low concentrations under four distinct conditions. The stability of the analyte was assessed under the following conditions: 24-h storage at 4 °C, three complete freeze–thaw cycles from −20 °C, 6-h bench-top stability at room temperature, and 6-h post-processing stability in the autosampler.

### 2.7. Liver Microsomes In Vitro

Rat liver microsomes (CYP450) were introduced into centrifuge tubes containing phosphate buffer. Different concentrations of the test compound were added to the experimental group, while an equal amount of buffer was added to the control group. Following a 10-min pre-incubation, NADPH was introduced to initiate the reaction, and the mixture was then incubated at 37 °C in a water bath for 1 h. The incubation system was composed of 10 μL of XMD12 substrate (0.5 μg/mL), 5 μL of rat liver microsomes (20 mg/mL), 20 μL of an NADPH generating system at a final concentration of 1 mM, and 465 μL of phosphate-buffered saline (pH 7.4). The NADPH generating system was prepared by mixing with 5 μL of a complementary Mix solution prior to use. This Mix solution contained MgCl_2_, EDTA, glucose-6-phosphate, glucose-6-phosphate dehydrogenase, and oxidized nicotinamide adenine dinucleotide phosphate (NADP^+^). At intervals of 0, 5, 15, 30, 45, 60, 90, and 120 min, the reaction was halted by adding a cold acetonitrile solution containing the internal standard at a 1:2 ratio. This mixture underwent vigorous vortexing for 3 min before being centrifuged at 14,000 rpm for 10 min. The resulting supernatant was dried using nitrogen gas and subsequently reconstituted with 50% acetonitrile. After thorough vortexing for 2 min, the solution underwent a second centrifugation at 14,000 rpm for 10 min, and the supernatant was then injected into LC-MS/MS for analysis.

### 2.8. Collection of Animal Specimens

#### 2.8.1. Plasma Exposure Study

Ten healthy SD rats were divided into two groups, each comprising five rats. Following a 12-h fasting period, the rats in the respective groups were administered XMD12 at dosages of 10 mg/kg via gastric gavage and 2 mg/kg via tail vein injection. The rats were anesthetized with isoflurane, and blood samples of approximately 150 uL were collected via the eye socket vein using capillaries. These samples were obtained prior to dosing (at 0 min) and at intervals of 0.083, 0.25, 0.5, 1, 1.5, 2.5, 4, 6, 8, 12 and 24 h post-dosing. The blood was then placed in centrifuge tubes pre-treated with heparin sodium, centrifuged at 3000 rpm for 10 min, and the resulting supernatant was stored in a refrigerator at −20 °C. The plasma samples underwent processing as per the outlined “Sample Preparation” methods and were analyzed using the specified procedures under “Instrument Conditions” to ascertain XMD12 concentration in the rat plasma at various time points. Utilizing DAS 3.2.8 (BioGuider Co., Shanghai, China), pharmacokinetic software facilitated data processing, computation of pertinent pharmacokinetic parameters, and the creation of blood concentration–time curves for the drug.

#### 2.8.2. Tissue Distribution Study

Following a 12-h fasting period, 25 SD rats were randomly divided into 5 groups, each comprising 5 rats. These rats were administered the drug at a dosage of 20 mg/kg through gastric gavage. Utilizing the pharmacokinetic drug concentration–time curves derived from plasma experiments, tissue samples from the five groups were collected at 0.5, 1, 2, 3 and 4 h post-dosing. Blood was drawn from the abdominal aorta to ensure minimal residual blood within the rats, followed by euthanasia. A variety of tissues including the heart, liver, spleen, lungs, kidneys, stomach, small intestine, fat, muscles, and brain were then harvested. After weighing, all samples were stored in a freezer at −80 °C. The subsequent processing and analysis followed the described methods, aiming to determine the concentration of XMD12 in each tissue sample at different time intervals.

#### 2.8.3. Urine and Feces Excretion Studies

Five male SD rats were housed individually in metabolic cages for adaptation for a day. During this period, urine and feces were collected as blank samples. The following day, the rats were orally administered XMD12 at a dose of 20 mg/kg by gastric gavage. Urine and feces samples were then collected at intervals of 0–4 h, 4–8 h, 8–12 h, 12–24 h, and 24–36 h post-administration. Measurements of volume and weight were recorded for each sample. Employing the outlined procedures, these samples underwent processing and analysis to determine the presence of XMD12 across different time spans within the collected urine and feces samples.

## 3. Results and Discussion

### 3.1. Method Validation

#### 3.1.1. Specificity and Carryover

**Figure 1:**
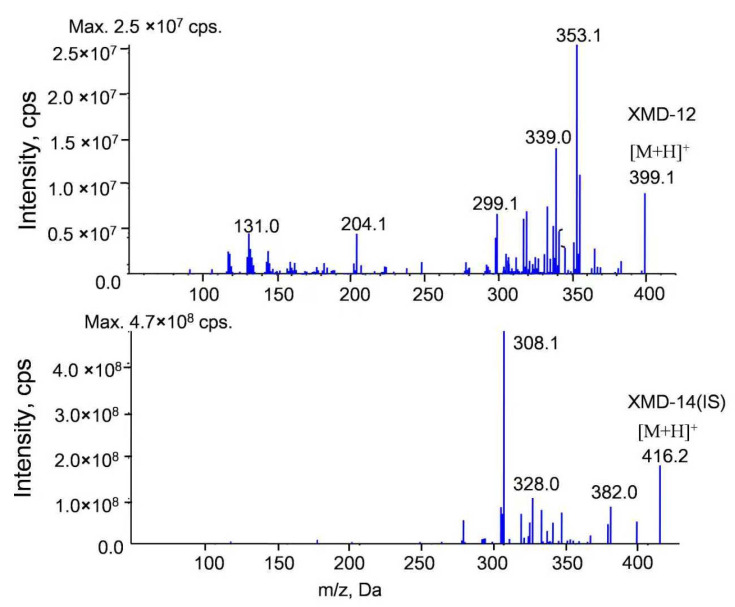
MS/MS spectra of XMD12 and IS (XMD14).

**Figure 2:**
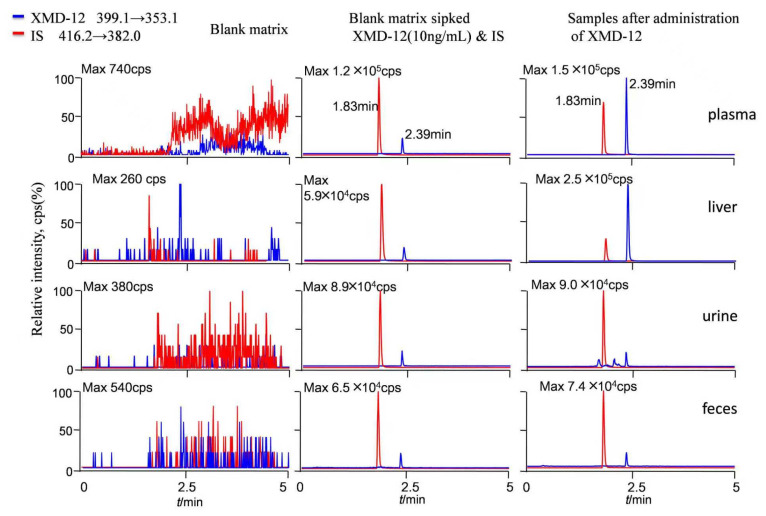
Representative LC-MS/MS chromatograms of XMD12 and IS (XMD14) in rat. Samples after administration of XMD-12: Plasma: 0.5 h after iv. 2.0 mg/kg XMD-12; Liver: 0.5 h after po. 20.0 mg/kg XMD-12; Urine: 0–4 h after po. 20.0 mg/kg XMD-12; Feces: 0–4 h after po. 20.0 mg/kg XMD-12.

The tandem mass spectrometry (MS/MS) spectra of XMD12 and its internal standard (IS), XMD14, are presented in Figure 1. Representative LC-MS/MS chromatograms of blank biological matrix, IS-spiked blank biological matrix, LLOQ samples, and rat samples are shown in Figure 2, demonstrating excellent specificity of the analytical method. Both XMD12 and IS were clearly separated without any interfering peaks. Consistent retention times were observed for XMD12 and IS across all tested matrices including plasma, liver microsomes, urine, feces, and tissue samples. The method showed no significant carry-over effects, with residual XMD12 peak areas in blank samples being less than 20% of those in LLOQ samples, and residual IS peak areas below 5% of the QC sample areas, confirming the precision and reliability of this analytical approach.

#### 3.1.2. Linearity

All biological samples demonstrated good linearity in their standard curves, with quantification ranges optimized according to matrix characteristics to balance matrix interference and sensitivity requirements. All analyses employed 1/*x*^2^ weighted least squares regression to correct heteroscedasticity and enhance quantitative accuracy at low concentrations. The corresponding regression equations, correlation coefficients, and quantification ranges are presented in Table 1.

#### 3.1.3. Precision, Accuracy and Stability

All biological matrices demonstrated satisfactory precision and accuracy in XMD12 detection. As exemplified by the representative results from plasma and liver tissue analyses (Table 2), both intra-day and inter-day precision showed relative standard deviations (RSD) below 10%, with accuracy ranging from 90.06% to 107.2%—fully compliant with FDA guidelines (RSD ≤ 15%, accuracy 85–115%). Subsequent stability testing under various conditions (storage temperature, duration, and freeze–thaw cycles) in these representative matrices (Table 3) revealed all RSD values below 10%. These results confirm that the developed method maintains high precision and accuracy in complex biological matrices while demonstrating excellent stability throughout sample storage and handling procedures, thereby fulfilling the comprehensive analytical requirements for pharmacokinetic studies.

#### 3.1.4. Extraction Recovery and Matrix Effect

The matrix effects of XMD12 across biological matrices are illustrated in Table 4, with matrix effect values (ME%) ranging from 87.05% to 109.37%, indicating comparable matrix interference across different biological samples. Furthermore, we evaluated the extraction recovery rates of XMD12. As demonstrated in plasma and liver tissue (Table 5), which were selected as representative matrices, the optimized sample pretreatment method achieved high and consistent recovery rates, establishing a stable and reproducible quantitative foundation for pharmacokinetic investigations.

### 3.2. Liver Microsomes In Vitro

**Figure 3:**
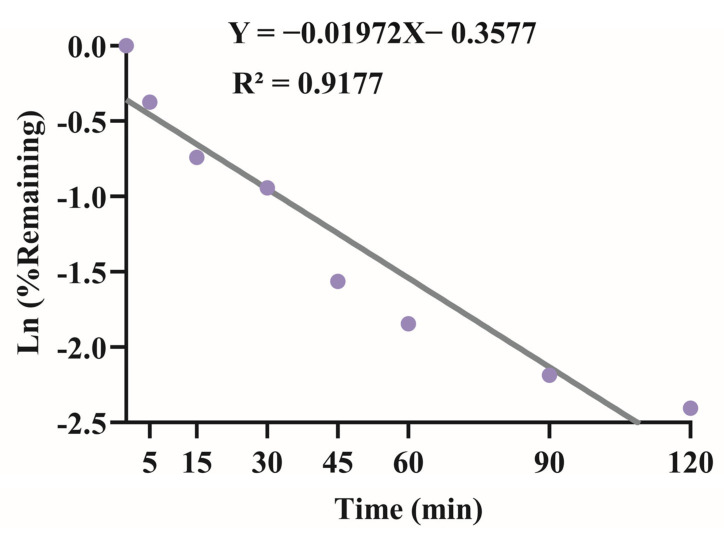
Linear regression of the natural logarithm of XMD12 remaining percentage versus incubation time in rat liver microsomes for intrinsic clearance (C_Lint_) determination.

We evaluated the metabolic stability of XMD12 using a rat liver microsome incubation system, with experimental results presented in Figure 3. XMD12 exhibited rapid hepatic metabolism, retaining 68.8% of the parent compound at 5 min, declining to 20% by 45 min, with only 13% remaining after 120 min of incubation. To quantitatively characterize this metabolic process, we performed regression analysis of the natural logarithm of the remaining percentage against incubation time, yielding the equation y = −0.01972x − 0.3577 (R^2^ = 0.9177). From this linear relationship, the degradation rate constant (*k*) was determined to be 0.0197 min^−1^. Applying the formula *CL_int_ = k × (V/P)*, where *V* represents the incubation volume (500 μL) and *P* denotes the total microsomal protein amount (0.1 mg), the intrinsic clearance was calculated as 98.5 μL/min/mg protein. This extensive biotransformation by hepatic enzymes (e.g., CYP450) observed in vitro aligns well with in vivo observations: the 87% metabolic rate at 120 min correlates with the 9% residual hepatic concentration measured at 5 h post-dosing, confirming sustained enzymatic clearance. These findings establish a coherent pharmacokinetic framework identifying hepatic metabolism as the predominant elimination mechanism. As a preliminary investigation preceding the plasma pharmacokinetic experiments, the liver microsome assay not only provided critical quantitative parameters but also elucidated the primary metabolic mechanisms underlying the observed first-pass effects, thereby establishing a mechanistic basis for interpreting subsequent in vivo pharmacokinetic behavior.

### 3.3. Pharmacokinetic Analysis and Bioavailability

**Figure 4:**
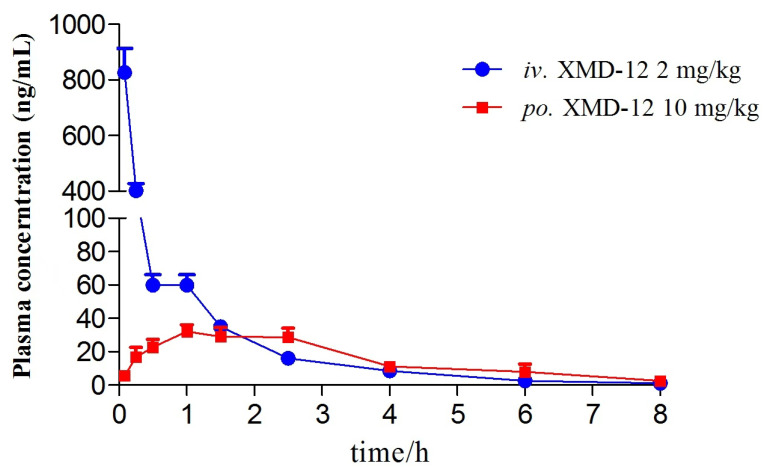
Plasma concentration–time curve of XMD12.

The plasma pharmacokinetic study employed high-performance liquid chromatography-tandem mass spectrometry (HPLC-MS/MS) to analyze the plasma pharmacokinetics of XMD12 in rats following single intravenous administration and gastric gavage. Mean plasma concentration–time profiles after a single dose (n = 5) are shown in Figure 4, with non-compartmental pharmacokinetic parameters summarized in Table 6. Following intravenous administration, XMD12 rapidly attained a peak plasma concentration (827 ng/mL) at 0.083 h, followed by a rapid decline to 59.88 ng/mL within 30 min, with concentrations falling below the lower limit of quantification (LLOQ) by 8 h. The compound displayed a short elimination half-life and high clearance, indicating a requirement for frequent dosing to sustain therapeutic concentrations. Mechanistically, the high intrinsic clearance (C_Lint_, 98.5 μL/min/mg protein) observed in liver microsomes accounts for the substantial systemic clearance observed in vivo, thereby explaining the potent first-pass effect following gastric gavage. The short plasma half-life (1 h) and high clearance observed here are fully consistent with the rapid degradation observed in liver microsomes (20% remaining at 45 min), confirming hepatic metabolism as the primary clearance pathway. Following gastric gavage, XMD12 showed slow absorption and markedly limited systemic exposure, achieving peak concentrations below 5% of intravenous levels and approaching the LLOQ within 8 h. The low bioavailability (6.67%) indicated significant first-pass metabolism during gastrointestinal absorption.

### 3.4. Tissue Distribution Study

**Figure 5:**
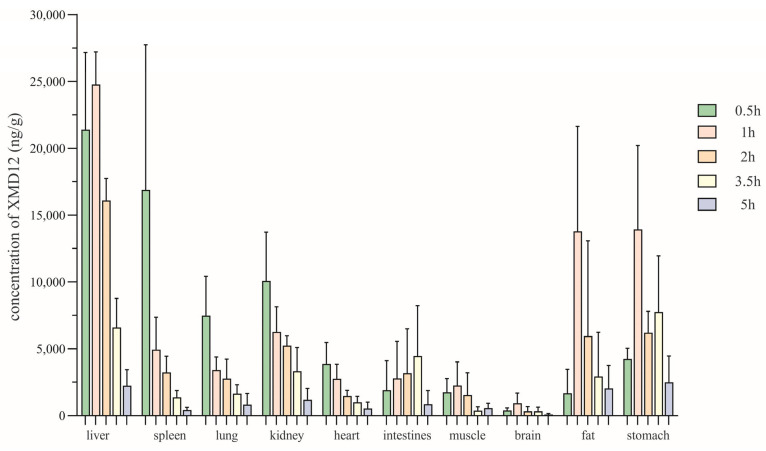
The tissue distribution at various time points after gastric gavage of XMD12 in SD rats.

To elucidate the tissue-level disposition underlying the observed pharmacokinetic behavior, we subsequently investigated the spatial and temporal distribution of XMD12 across major organs. Based on pharmacokinetic findings, tissue distribution was assessed at 0.5, 1, 2, 3.5, and 5 h post-dosing. XMD12 showed rapid and extensive tissue distribution following gastric gavage (20 mg/kg), as depicted in Figure 5. The extensive tissue distribution observed in rats represents a characteristic shared with marketed CDK4/6 inhibitors, as reflected by the large volume of distribution (Vd) reported in humans for palbociclib (~2800 L) and ribociclib (Vd = 1090 L) [14,15]. The liver was identified as the primary site of metabolism, reaching a peak concentration (24,768 ± 2436.62 ng/g) at 1 h followed by a progressive decline to 9% of the peak value (2241.9 ± 1193.07 ng/g) by 5 h, indicating preferential hepatic accumulation and rapid clearance via metabolic and biliary pathways. Gastrointestinal tissues (stomach and intestines), serving as absorption sites, reached peak drug concentrations within 0.5–1 h, suggesting efficient mucosal absorption into systemic circulation. The pronounced kidney accumulation concurrent with minimal excretion (0.0068%) underscores complex renal disposition. This profile is indicative of substantial tubular reabsorption, potentially involving OAT/OCT transporters, a hypothesis that warrants further investigation. A detectable brain concentration (930.17 ± 762.76 ng/g) at 1 h indicated limited blood–brain barrier penetration. All tissues exhibited concentrations below 10% of peak levels by 5 h, suggesting low potential for in vivo accumulation.

### 3.5. Excretory Study

**Figure 6:**
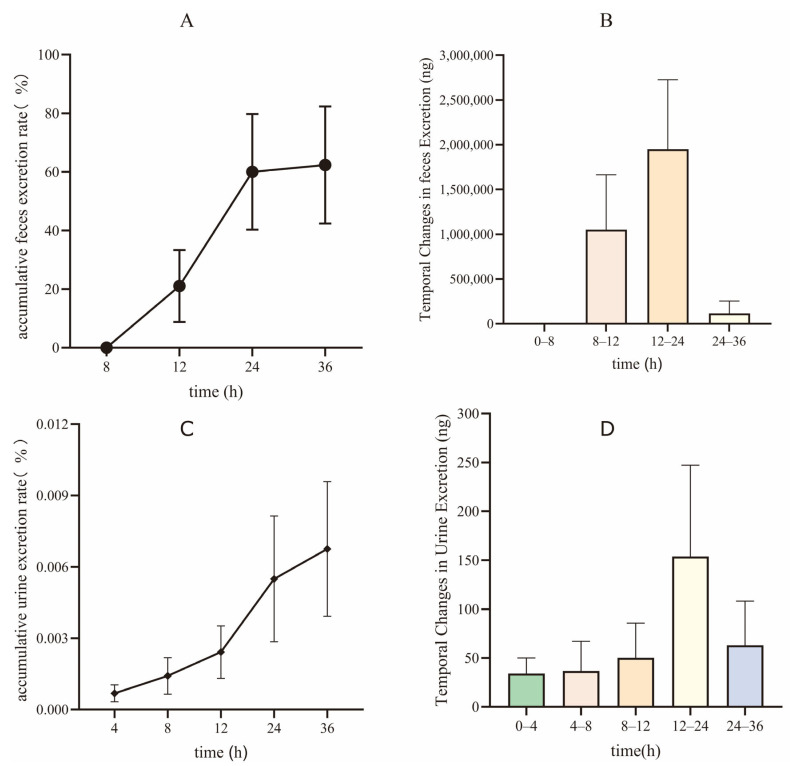
Excretion of XMD12 in SD rats. (**A**) accumulative feces excretion rate; (**B**) temporal changes in feces Excretion; (**C**) accumulative urine excretion rate; (**D**) temporal changes in urine excretion.

Having delineated the compound’s distribution pattern, we next focused on its elimination pathways to complete the overall ADME profile. Figure 6 illustrates the excretion dynamics of XMD12 in rats following gastric gavage, demonstrating fecal dominance as the primary elimination route. Cumulative fecal excretion accounted for 62.36% ± 19.97% of the administered dose, exhibiting a distinct time-dependent profile with rapid elimination occurring between 12–24 h followed by a sharp decline (only trace amounts detected at 24–36 h), indicating efficient intestinal clearance and/or metabolic conversion. The fecal-dominated excretion pattern directly reflects the extensive hepatic accumulation and rapid clearance observed in tissue distribution studies, thereby completing the ADME pathway from absorption to elimination. The predominant fecal excretion observed in rats (62.36%) is consistent with the principal elimination route reported for all approved CDK4/6 inhibitors in humans (palbociclib: 74.1%; ribociclib: 69.1%; abemaciclib: 81% fecal excretion) [14,15], strongly supporting a class-specific preference for hepatobiliary elimination over renal excretion. Urinary excretion remained negligible (0.0068% ± 0.0028% cumulative rate), despite showing a similar peak elimination window (12–24 h), confirming the minor role of renal elimination. Together with pharmacokinetic data (low bioavailability: 6.67%) and hepatic distribution profiles (peak liver concentration: 24,768 ng/g), these results corroborate that orally administered XMD12 undergoes extensive hepatic metabolism followed by biliary-mediated fecal elimination.

## 4. Conclusions

This study established and validated a simple, rapid HPLC-MS/MS method for quantitative analysis of XMD12 in rat plasma, liver microsome preparations, tissues, urine, and feces, providing a foundational analytical platform for subsequent investigations of related compounds. The key pharmacokinetic characteristics revealed (1) delayed absorption with significantly limited systemic exposure following gastric gavage; (2) preferential hepatic accumulation post-intestinal absorption followed by rapid metabolic/biliary clearance, complemented by collaborative elimination through other organs; and (3) time-dependent cumulative fecal excretion (62.36% ± 19.97%) as the dominant elimination pathway, with metabolites undergoing terminal clearance via biliary–intestinal excretion. These systematic findings elucidate XMD12’s dynamic disposition pattern of “restricted absorption–hepatic metabolic priority–multi-tissue collaborative clearance”, highlighting the need for structural modifications (e.g., incorporation of metabolically stable moieties) or combination strategies with CYP enzyme inhibitors to circumvent first-pass effects and enhance the therapeutic window.

## Figures and Tables

**Table 1:** Standard calibration curves and LLOQ of XMD12 in different matrices (n = 3).

Matrix	Equation (1/*x*^2^, Weighting Index)	Range (ng/mL)	LLOQ/(ng/mL)
Plasma	y = 0.00306x + 0.00342 (r = 0.9985)	2.5–1000	2.5
Liver	y = 316x + 21,300 (r = 0.9936)	10–2000	10
Spleen	y = 400x + 1710 (r = 0.9919)	10–2000	10
Heart	y = 374x + 507 (r = 0.9955)	10–2000	10
Kidney	y = 379x + 1370 (r = 0.9989)	10–2000	10
Lung	y = 375x + 1490 (r = 0.9959)	10–2000	10
Brain	y = 459x + 399 (r = 0.9924)	10–2000	10
Muscle	y = 414x + 2710 (r = 0.9954)	10–2000	10
Stomach	y = 485x + 4640 (r = 0.9931)	10–2000	10
Fat	y = 439x + 965 (r = 0.9924)	10–2000	10
intestines	y = 453x + 1250 (r = 0.9946)	10–2000	10
Urine	y = 0.00582x + 0.00438 (r = 0.9963)	2.5–1000	2.5
Feces	y = 0.00662x + 0.00437 (r = 0.9952)	2.5–1000	2.5
Microsome	y = 1200x – 7790 (r = 0.9952)	100–2000	100

**Table 2:** Intra- and inter-day precision and accuracy for XMD12 in rat plasma and liver.

Matrix	Nominal Concentration (ng/mL)	Intra-Day (n = 5)			Inter-Day (n = 15)		
		Measured Concentration (ng/mL)	Precision (RSD,%)	Accuracy (%)	Measured Concentration (ng/mL)	Precision (RSD,%)	Accuracy (%)
Plasma	2.5	2.49 ± 0.23	9.20	99.76	2.41 ± 0.21	8.80	96.22
	5	5.35 ± 0.31	5.80	107.20	5.08 ± 0.37	7.38	101.76
	90	83.70 ± 2.67	3.19	93.00	86.96 ± 8.37	9.63	96.68
	800	733.60 ± 11.01	1.50	91.74	772.27 ± 69.39	8.99	96.56
Liver	30.0	29.5 ± 2.6	8.95	98.3	30.0 ± 2.2	7.43	100.05
	800.0	802.8 ± 27.9	3.48	100.4	815.1 ± 40.8	5.00	101.84
	1600.0	1582.5 ± 92.5	5.85	90.06	1582.2 ± 87.6	5.54	95.85

**Table 3:** The stability of XMD12 in various biological matrices.

Matrix	Nominal Concentration (ng∙mL^−1^)	6 h at Room Temperature		Freeze–Thaw (3 Circles)		15 d,−80 °C (15 Days and −80 °C)		Autosample (at Ambient Temperature for 6 h)	
		Precision (%, RSD)	Accuracy (%)	Precision (%, RSD)	Accuracy (%)	Precision (%, RSD)	Accuracy (%)	Precision (%, RSD)	Accuracy (%)
Plasma	5	6.46	105.58	8.12	97.10	3.93	94.20	5.22	92.16
	90	2.35	108.60	6.38	107.22	5.81	93.76	3.06	92.44
	800	5.05	110.80	5.18	106.80	5.72	102.22	4.39	90.02
Liver	30.0	3.67	100.93	6.44	100.47	1.61	97.53	3.01	100.80
	800.0	4.28	101.93	3.69	99.78	3.12	99.40	2.70	103.;10
	1600.0	5.72	101.56	7.64	97.75	5.70	101.63	6.12	100.94

**Table 4:** Matrix effect of XMD12 in various biological matrices.

Matrix	Nominal Concentration (ng/mL)	Matrix Effect (%, Mean)	RSD (%)
Plasma	5	93.93 ± 7.07	7.53
	800	92.87 ± 4.61	4.96
Liver	30	104.17 ± 7.22	6.93
	1600	94.03 ± 5.50	5.85
Spleen	15	101.13 ± 4.81	4.76
	1600	100.37 ± 5.88	5.86
Heart	30	92.28 ± 3.78	4.09
	1600	87.05 ± 3.57	4.10
Kidney	30	96.21 ± 6.22	6.46
	1600	91.88 ± 4.37	4.76
Lung	30	90.56 ± 2.53	2.79
	1600	94.83 ± 4.53	4.78
Brain	30	87.33 ± 1.36	1.56
	1600	91.92 ± 2.40	2.61
Muscle	30	102.21 ± 5.32	5.20
	1600	101.31 ± 6.37	6.29
Stomach	30	91.38 ± 3.85	4.21
	1600	97.06 ± 6.87	7.08
Intestines	30	107.38 ± 5.83	5.43
	1600	109.37 ± 3.74	3.42

**Table 5:** Extraction recovery of XMD12 in various biological matrices.

Matrix	Nominal Concentration (ng∙mL^−1^)	Mean ± SD (%)	RSD (%)
Plasma	5	84.52 ± 9.46	11.19
	90	87.99 ± 4.79	5.45
	800	88.27 ± 7.47	8.46
Liver	30	90.25 ± 6.07	6.73
	800	95.17 ± 5.36	5.63
	1600	94.26 ± 3.14	3.33

**Table 6:** Pharmacokinetic parameters of XMD12 (Mean ± SD, n = 5).

Parameters	i.v. (2 mg∙kg^−1^)	p.o. (10 mg∙kg^−1^)
Cmax (ng∙mL^−1^)	827.00 ± 191.30	38.26 ± 10.90
Tmax (h)	0.083	1.300 ± 0.758
AUC0-t (h∙ng∙mL^−1^)	357.21 ± 66.60	117.78 ± 28.97
AUC0-∞ (h∙ng∙mL^−1^)	360.03 ± 66.00	120.06 ± 30.19
t1/2 (h)	1.47 ± 0.29	1.21 ± 0.16
MRT0-t (h)	0.74 ± 0.08	2.475 ± 0.344
CL (L∙h^−1^∙kg^−1^)	5.69 ± 0.95	86.64 ± 16.72
V (L∙kg^−1^)	12.29 ± 3.98	150.59 ± 34.37
F (%)	-	6.67

## Data Availability

The data that support the findings of this study are available from the corresponding author upon request.

## Supplementary Materials

The following supporting information can be downloaded at: https://www.mdpi.com/article/10.3390/pharmaceutics17121545/s1, Figure S1: The 1H-NMR of the compoundXMD12, Figure S2: The 13C-NMR of the com-poundXMD12.
