# Supplementary material for: The Absorption, Distribution, Excretion, and In Vitro Hepatic Microsomal Metabolism of the Novel CDK Compound XMD12 in Sprague-Dawley Rats
Source: Pharmaceutics. 2025 Nov 30;17(12):1545. doi: 10.3390/pharmaceutics17121545 (PMC12736570; doi:10.3390/pharmaceutics17121545)
Supplement: Supplementary file 1 [file pharmaceutics-17-01545-s001.zip › pharmaceutics-3967082-supplementary.pdf]

pharmaceutics

MDPI

Article

The Absorption, Distribution, Excretion, and In Vitro Hepatic Microsomal Metabolism of the Novel CDK Compound XMD12 in Sprague-Dawley Rats

Xue-Hai Zheng [1], Yan-Chun Chang [1,2,3,4], Yong-Hui Li [1,2,3,4], Yu-Xia He [1], Pei-Xiong Zhao [1], Fei-Fei Wang [1], Jun-Yu Xu [1,2,3,4,]* and Yin-Feng Tan [1,2,3,4,]*

1. School of Pharmacy, Hainan Medical University, 3 Xueyuan Road, Haikou 571159, China; xuehaihaihai@gmail.com (X.-H.Z.); 18419870189@163.com (Y.-C.C.); lyhssl@126.com (Y.-H.L.); 15738088269@163.com (Y.-X.H.); zhaopeixiong0824@163.com (P.-X.Z.); 15799016982@163.com (F.-F.W.)
2. Engineering Research Center of Tropical Medicine Innovation and Transformation of Ministry of Education, Haikou 571199, China
3. Hainan Key Laboratory for Research and Development of Tropical Herbs, School of Pharmacy, Hainan Medical University, Haikou 571199, China
4. Haikou Key Laboratory of Li Nationality Medicine, Haikou 571199, China

* Correspondence: xujy201309@muhn.edu.cn (J.-Y.X.); hy0207059@hainmc.edu.cn (Y.-F.T.); Tel./Fax: +86-898-6689-0907 (J.-Y.X. & Y.-F.T.)

Supplementary Materials

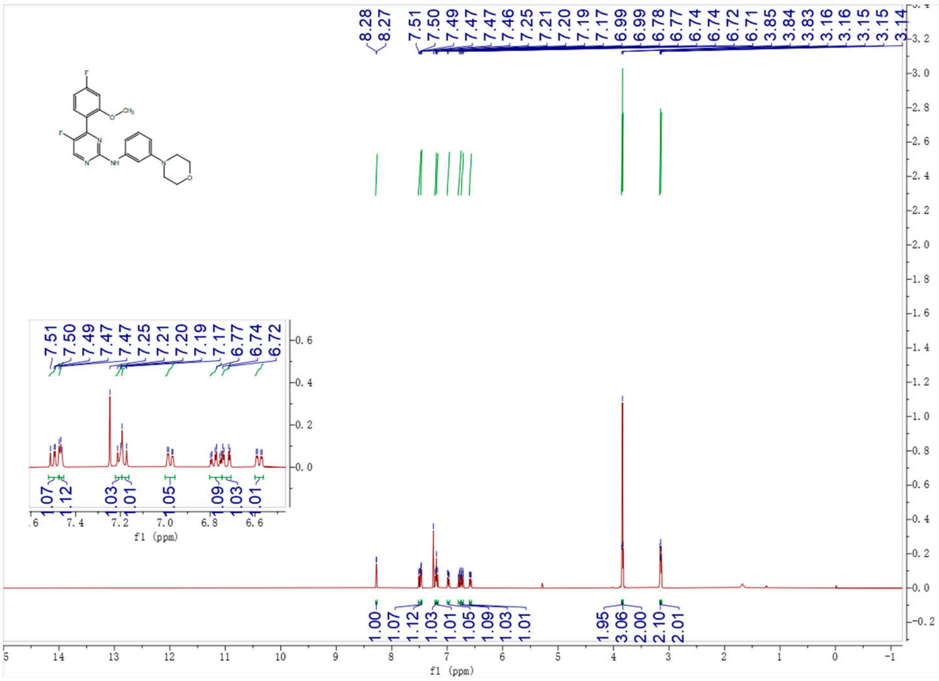

Figure S1: The 1H-NMR of the compound XMD12.

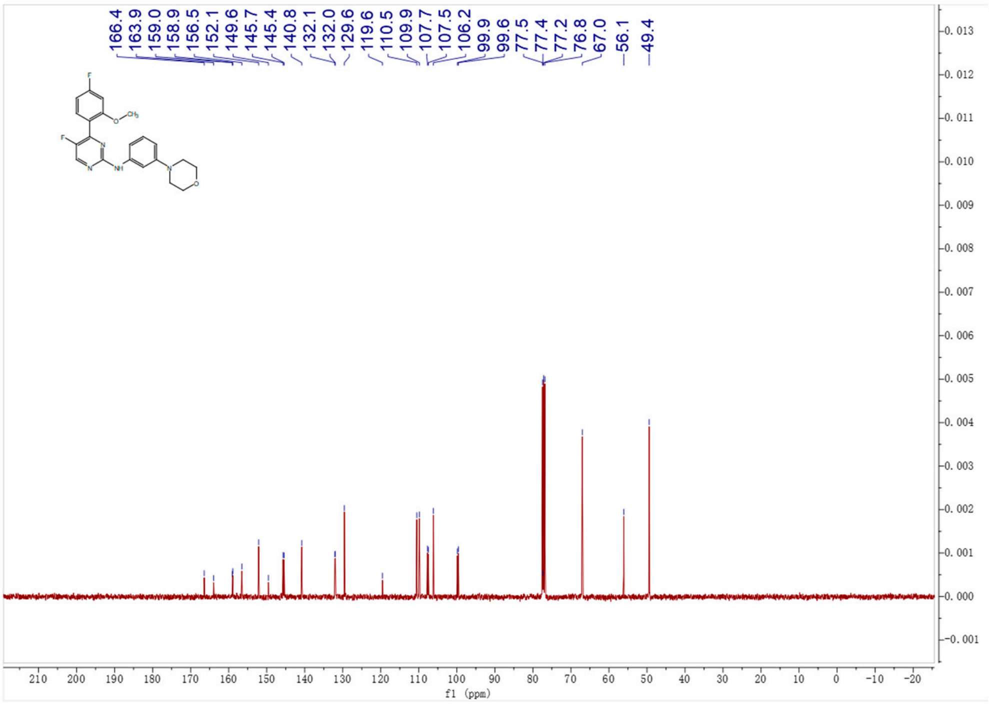

Figure S2: The 13C-NMR of the compoundXMD12.
